# Effect of a Guide-Suture-Assisted Modified Fascial Closure Technique on Postoperative Pain and Early Mobilization After Cesarean Section: A Mixed-Methods Study

**DOI:** 10.3390/healthcare14070972

**Published:** 2026-04-07

**Authors:** Fatma Kılıç Hamzaoğlu, Betül Dik, Emine Türen Demir, Hasan Energin

**Affiliations:** 1 Department of Gynecology and Obstetrics, Faculty of Medicine, Necmettin Erbakan University, 42080 Konya, Türkiye; eturen1@hotmail.com (E.T.D.); hasanenergin@gmail.com (H.E.); 2 Department of Gynecology and Obstetrics, Zonguldak Maternity and Children’s Hospital, 67030 Zonguldak, Türkiye; dr.betuldik@hotmail.com

**Keywords:** cesarean section, postoperative pain, fascial closure, early mobilization, mixed-methods study

## Abstract

Background/Objections: One of the most common surgical procedures performed internationally is the cesarean section. It is known to be associated with intense postoperative pain and a slow recovery process. Focusing on surgical techniques, especially the type of fascial closure, is an area that has received very little attention when it comes to postoperative pain and rapid recovery. Using a mixed-methods approach, the primary objective of this study was to assess the impact of guide-suture-assisted modified fascial closure on postoperative pain and early mobilization after cesarean sections. Methods: Women undergoing elective cesarean sections with Pfannenstiel’s incision were the study participants of this prospective, single-center, randomized mixed-methods study. Participants were enrolled in the study and randomized to either classical continuous fascial closure or guide-suture-assisted modified fascial closure, which was carried out in a 1:1 ratio. Quantitative data assessed postoperative pain through the Visual Analog Scale (VAS), a Numeric Rating Scale (NRS), and the Short-Form McGill Pain Questionnaire (SF-MPQ), and functional recovery was assessed through walking distances at postoperative 6, 12, 24, and 48 h. Qualitative data were collected via semi-structured interviews and analyzed through conventional content analysis to understand the patients’ perceptions of pain and recovery experiences. Results: The first 24 h postoperative period pain levels were significantly lower for the modified fascial closure group versus the classical closure group (*p* < 0.05). Moreover, the modified closure group had a significantly better functional recovery, evidenced by walking greater distances at 12, 24, and 48 h postoperative. Qualitative results indicated improved comfort and stronger early mobilization confidence, in addition to less movement apprehension, consistent with the above results, among those with the modified technique. Conclusions: The modified fascial closure technique with guide suture was linked to less pain in the early postoperative period and better functional recovery after cesarean section. This technique is a good candidate for addition to standard obstetric procedures since it is cost effective, easily added, and surgical practice will improve comfort for mothers and assist with early mobilization.

## 1. Introduction

Globally, one of the most commonly performed surgical procedures is cesarean section, and its incidence has increased steadily over the past decades. The World Health Organization states that, currently, one in five births is via cesarean section, and this ratio is likely to continue increasing, especially in countries with middle and high incomes [1]. While cesarean deliveries is a life-saving intervention when warranted, there is a considerable amount of postoperative morbidity. Issues related to pain, delayed mobility, longer recoveries, and increased healthcare utilization are of particular concern.

Following surgical procedures, postoperative pain is a major clinical issue. Inadequate pain control negatively affects maternal comfort, mobility, breastfeeding, and mother–infant interaction. From an anesthetic-surgical outlook, the better postoperative analgesia, the better the respiratory function, the lower the risk of a thromboembolic event, and the quicker the person is to recover functionally [2]. After the delivery of a baby via cesarean section, many women have reported suffering from acute pain, and a smaller percentage go on to experience chronic pain [3]. Therefore, in order to be successful, any strategies implemented to address pain control should be a top concern in this area of surgical obstetrics.

The types of surgical technique employed will influence how the patient perceives pain postoperatively. The way in which the abdominal wall and fascial layers are closed, in addition to the type of incision made, can influence the negative consequences of closure such as wound tension, tissue ischemia, and inflammation. These can all contribute to the negative experience a patient can have after surgery, as well as the effects the surgery and recovery will have on them [4]. Meta-analyses addressing techniques for fascial closure have shown that while there are not any significant differences in the overall quality of the wound between the different closure techniques, minor differences have the potential to result in changes to complications and how patients feel after surgery. Similarly, randomized studies have focused on the differences between interrupted and continuous techniques of fascial closure and have shown differences in the negative consequences of the wound, such as dehiscence and pain [5,6].

Some closure techniques, such as the Smead–Jones method, are claimed to enhance the strength of the fascial closure while reducing the line of mechanical stress. Studies that compared midline laparotomy have shown that interrupted techniques or modified Smead–Jones techniques have potential advantages over the conventional continuous closure technique regarding post-operative results and wound closure, and how this affects the patient post-operatively. These techniques have primarily been assessed in general surgery and emergency surgery, but the principles behind them, which are the improved alignment of fascial edges and the reduction in localized tension, also apply to cesarean section surgery [7,8,9].

Even though there is a lot of literature regarding techniques for a cesarean section, most studies focus on incision type, utero closure, or skin closure, while the impact of modifications of fascial closure on pain and functional recovery has been largely ignored [10,11]. In addition, most studies focus on qualitative pain scaring, which do not encapsulate the full range of the pain suffering experience.

This study aimed to analyze the impact of a guide suture-assisted modified fascial closure technique on postoperative pain and functional recovery after cesarean section using a mixed method design. While patients’ pain intensity and recovery were analyzed quantitatively, their pain and recovery experiences were studied qualitatively. Compared to the conventional method, the modified closure technique produced lower early postoperative pain scores, improved mobilization, and better pain score of the patients. With the combination of patient-centered and clinical outcomes, this study demonstrated the importance of incorporating a low-cost surgical technique to enhance patient comfort and recovery, proving the value of such techniques in everyday obstetric practice.

## 2. Materials and Methods

This study was conducted using an explanatory sequential mixed-methods design as a prospective, single-center, parallel-group, randomized controlled trial with an embedded qualitative component. In this approach, quantitative data were first collected and analyzed to assess postoperative pain, functional recovery, and analgesic requirements after cesarean section; subsequently, qualitative data were obtained to explain and contextualize the quantitative findings. The study focused on comparing one of the conventional continuous fascial closure techniques with the guide-suture-assisted modified fascial closure technique. The study was conducted between October 2025 and January 2026. This study was approved by the Necmettin Erbakan University Ethics Committee for Non-Drug and Non-Medical Device Research (decision no: 2025/6054; meeting date: 24 October 2025), and all procedures were conducted in accordance with the Declaration of Helsinki.

This study was conducted in the Department of Obstetrics and Gynecology, Faculty of Medicine, Necmettin Erbakan University. Participants were women aged between 18 and 35 years who were scheduled to have an elective cesarean section with a Pfannenstiel incision. Participants were recruited consecutively from women undergoing elective cesarean delivery who met the inclusion criteria during the study period. The inclusion criteria were the patient’s single gestation, at least 37 weeks of gestation, and the American Society of Anesthesiologists (ASA) physical status class I or II. Participants were excluded if they were to have an emergency cesarean section, and if they had a history of previous cesarean sections, severe intra-abdominal adhesions, coagulopathy, or an active infection (and) intraoperative complications. Prior to study enrollment, all participants signed a written informed consent form. All procedures were performed under standardized spinal anesthesia.

Quantitative sample (trial population): A total of 60 participants were enrolled and randomized, with 30 allocated to the classical continuous fascial closure group and 30 allocated to the guide-suture-assisted modified fascial closure group.

Qualitative sample (interview population): Semi-structured interviews were conducted with a purposive subsample of participants (n = 12), selected from both trial arms (classical group: n = 6; modified group: n = 6) to ensure maximum variation in pain intensity and recovery experiences (e.g., low vs. high pain scores and early vs. delayed mobilization). Recruitment to the qualitative arm continued until thematic saturation was achieved. The qualitative subsample (n = 12) was selected after preliminary quantitative analysis to ensure maximum variation sampling (e.g., different pain intensities and mobilization outcomes). Not all participants were included in the qualitative phase due to methodological considerations typical of mixed-methods designs, where in-depth exploration is prioritized over representativeness. This approach minimized redundancy and allowed thematic saturation to be achieved.

Using the sealed-envelope randomization technique, trial participants were randomized in a 1:1 ratio to one of the two groups. Group allocation was conducted right before surgery to avoid selection bias. One group experienced classical continuous fascial closure, while the other group experienced the guide-suture-assisted modified fascial closure technique.

To eliminate inter-operator bias, the same skilled surgeon performed all the surgical procedures. All surgeries were conducted under spinal anesthesia, and a Pfannenstiel skin incision was used for all surgeries. Between groups, techniques for skin closure and uterine closure were standardized, and the peritoneum was not closed for any of the patients.

In the classical fascia closure group, one surgeon would start at one end of the incision and would end at the stitch the opposite end while performing closure with a continuous suture technique using Vicryl 1-0 (polyglactin 910). In the modified fascia closure group, prior to starting the continuous closure, a guide suture with Vicryl 1-0 was placed at the corner, which was intended to be the end of the suture line. This guide suture ensured that the edges of the fascia were aligned properly and reduced the chances of the rectus muscles being sutured inadvertently. Once the guide suture was placed, the fascial closure was performed using the continuous suture closure technique. Other than this modification, all other surgical procedures were the same between the two groups.

The assessment of postoperative pain and functional recovery employed quantitative tools of evidence-based value. Pain was measured at 6, 12, 24, and 48 h post-surgery using a 0–10 Visual Analog Scale (VAS) at each time point. Pain with movement was measured using a Numeric Rating Scale (NRS) and descriptions of pain (e.g., burning or stabbing) were collected using a Short-form McGill Pain Questionnaire (SF-MPQ). Functional recovery was assessed by recording how far each patient was able to walk at each time point. Also, the type and frequency of each participant’s pain medication were recorded over the first 48 h post-surgery.

*Qualitative Data Collection*

After collecting the quantitative data, the qualitative data were obtained with semi-structured face-to-face interviews conducted in the early postoperative period. The early postoperative period was defined as the first 24 h following surgery. Participants were selected from each surgical group to ensure a diversity of experiences with postoperative pain and recovery. The interviews focused on the assessment of pain (how much of it was there? What type of pain was there?), the ability to move, the confidence in moving, obtaining the right amount of pain medication, and the recovery experience as a whole. With the participants’ approval, the interviews were recorded and transcribed.

*Qualitative Data Analysis*

As for the qualitative data, the transcripts were analyzed using conventional content analysis, which involves repeated reading to achieve data immersion. Meaningful units of text were coded, utilizing inductive methods, and these codes were consolidated and grouped into categories. Subsequently, the categories were systemized into primary themes and subthemes pertaining to the categories and broad features of the data. These themes and subthemes captured the data inter patterns and phenomena of pain, the experience of mobilization, and perceptions of recovery. To add to the analysis, the data were supplemented with extracts of texts that best represented the themes.

*Statistical Analysis*

SPSS software version 25.0 (IBM Corp., Armonk, NY, USA) was used to conduct the statistical analysis. The data were evaluated for distribution, and both the descriptive and the graphical methods were used for analysis. The continuous variables were expressed in terms of with mean ± standard deviation or median (25th–75th) percentile, and these values were used to assess the distribution of the values. For comparative analysis, every postoperative time frame was treated independently. For the analysis of continuous variables, the Mann–Whitney U test was used, and the dependent variables were treated as categorical, and these were expressed as frequency counts and percentages (%), for which the chi-square test was applied. For each of the analysis, a significance level of 0.05 was used to determine statistical significance.

*Integration of Quantitative and Qualitative Data*

Combining qualitative and quantitative results took place during the interpretation process. In this case, qualitative results contributed to the explanation and enhancement of the quantitative results, especially related to pain perception, mobilization behavior, and the use of analgesics, which together provided a holistic understanding of postoperative recovery from the perspective of the patients.

*Ethical Considerations*

The study was conducted in accordance with the Declaration of Helsinki and approved by the Necmettin Erbakan University Ethics Committee for Non-Drug and Non-Medical Device Research (decision no: 2025/6054; meeting date: 24 October 2025). All actions taken were consistent with the Declaration of Helsinki. All participants provided written informed consent before participation in the study.

**Figure 1:**
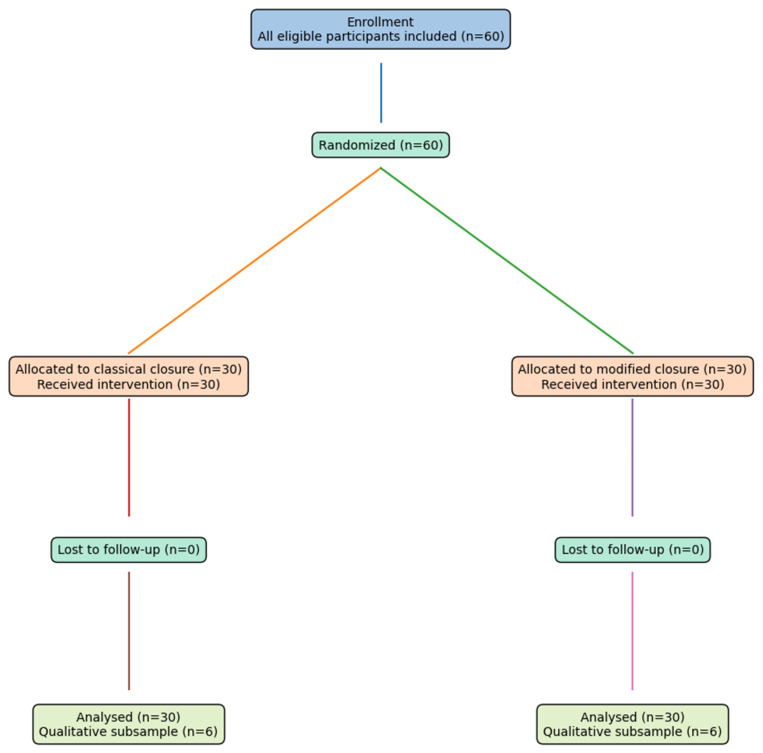
CONSORT Flow Diagram.

*Trial Registration:* This study was registered at ClinicalTrials.gov (Registration Number: NCT07436520) (Figure 1).

## 3. Results

The findings presented in Table 1 demonstrate that the two surgical technique groups were comparable in terms of demographic and baseline clinical characteristics. The mean age was 25.33 ± 3.86 years in the classical fascia closure group and 26.83 ± 4.12 years in the modified fascia closure group, with no statistically significant difference between the groups (*p* = 0.152), indicating similar age distributions.

Similarly, the mean body mass index (BMI) values were 27.51 ± 1.39 kg/m^2^ in the classical group and 27.43 ± 1.50 kg/m^2^ in the modified group, with no statistically significant difference observed (*p* = 0.825). These results suggest that both groups were comparable with respect to BMI.

No significant difference was observed between the groups in terms of time to first mobilization. The median mobilization time was 6 h in both groups, with an interquartile range of 5–8 h (*p* = 0.256), indicating that the surgical technique did not significantly influence early mobilization time (Table 1).

The results presented in Table 2 indicate marked differences in postoperative pain levels and functional recovery between the two fascia closure techniques. Analysis of VAS, NRS, and SF-MPQ scores revealed that pain levels were significantly higher in the classical fascia closure group during the early postoperative period, particularly at the 6th, 12th, and 24th postoperative hours. These differences were statistically significant for VAS and NRS scores at 6, 12, and 24 h (all *p* < 0.05), as well as for SF-MPQ scores at the same time points (all *p* < 0.05).

In contrast, patients in the modified fascia closure group reported consistently lower pain scores during these early postoperative intervals. When resting and ambulation-related VAS scores were evaluated together, significantly lower pain levels were observed in the modified technique group at the 6th, 12th, and 24th hours (all *p* < 0.05). However, no statistically significant difference between the groups was detected at the 48th postoperative hour (*p* > 0.05).

Regarding functional recovery, walking distance was significantly greater in the modified fascia closure group at the 12th, 24th, and 48th postoperative hours (*p* = 0.007 at 12 h; *p* < 0.001 at 24 and 48 h), indicating earlier and more effective mobilization in this group. At the 6th postoperative hour, no statistically significant difference in walking distance was observed between the groups (*p* = 0.114).

Overall, these findings suggest that the modified fascia closure technique is associated with lower postoperative pain levels and faster functional recovery in the early postoperative period compared with the classical technique, with these advantages supported by statistically significant results (Table 2).

Postoperative analgesic requirements were compared between the two surgical techniques, and the frequency of use for each analgesic agent is presented in Table 3. Analgesic use rates were analyzed using the chi-square test, with *p* < 0.05 considered statistically significant.

Significant differences were observed between the groups with respect to postoperative analgesic consumption. The use of dexketoprofen trometamol (50 mg, intravenous) was significantly higher in the modified fascial closure group (*p* < 0.001). In contrast, diclofenac sodium (75 mg, intramuscular) and tenoxicam (20 mg, intravenous) were used significantly more frequently in the classical fascial closure group (*p* < 0.001 and *p* = 0.012, respectively). Similarly, the use of tramadol hydrochloride (100 mg, intravenous) was significantly higher in the classical group compared with the modified group (*p* = 0.006). No statistically significant difference was observed between the groups regarding the use of 1000 mg (intravenous) paracetamol (*p* = 0.830) (Table 3).

Content analysis revealed the major themes and themes associated with the differences in pain perception and the experiences of mobilization and recovery pertaining to both surgical techniques, accompanied by the quotations of the representative participants to substantiate the themes provided (Table 4).

The preeminent qualitative findings contextualize the pain and recovery associated with the different closure techniques of fascia, the mapping of the different closure techniques of fascia as the center of the conflicting patterns of pain and recovery. Patients associated with the closure of fascia by surgical means in the classical fashion felt pain as if sharpened, burned, or stabbed, and expressed their fears pertaining to the early mobilization. These perceptions of pain and the statements made by the patients correlate and justify the increased scores of VAS, NRS, and SF-MPQ that presented in the initial stages of the post-operative period by the patients.

Conversely, patients with modified fascia closure techniques were considerably pain free and described their pain in vague terms, as pressure, or even tolerable, and of blunt pain. These patients also reported a heightened confidence in their ability to walk and a lesser felt dependence on the strong pain relievers. These qualitative insights illustrate the significantly lower pain scores and the better-performing walking distance measurements at 12, 24, and 48 h after surgery.

## 4. Discussion

In the past decade, the number of common surgical operations being performed across the globe has steadily risen, with cesarean sections leading the way in terms of frequency [1]. Even though the practice of cesarean section can be lifesaving for the mother and baby in certain critical situations, it does come with a number of risks and complications, particularly in the post-operative phase of care, which includes having to endure a prolonged recovery, increased healthcare costs, and issues with delayed ambulation [1,10,11]. Maternal discomfort is the most immediate result of post-surgical complications, and it is for this reason that post-operative cesarean pain has been recognized as a major clinical issue, in addition to the problems that it can create in mother–infant bonding, postpartum interactions, maternal ambulation, and breastfeeding, as well as in the development of chronic pain syndromes [2,3].

It has been demonstrated that a myriad of explanations exists for the intense pain endured after a surgical procedure, including, among other risks, the inadequate management of postoperative analgesic therapy, the surgical procedures performed, the anesthetics used, and the extent of the tissue trauma sustained [2,12]. Jin and colleagues showed that persistent post-surgical pain is a common occurrence after a cesarean section and that the most important risk factor for it is pain severity immediately after the surgical procedure [3]. From this, it is evident that there is a clinical need to limit the amount of postoperative pain experienced in order to help avoid the development of chronic pain syndromes in the future.

Excessive tension of wound margins, poor alignment of fascial surfaces, and other factors such as ischemia and inflammation can increase nociceptive signaling and, therefore, increase postoperative pain at the incision site [4,5,6]. Cançado et al. point out that aside from anesthetic approaches, surgical variables meritoriously affect the acute and chronic pain aftermath of a cesarean section [12]. Hence, in this light, we believe that fascial closure mechanics are an unappreciated, adjustable, and, importantly, impactful surgical aspect related to postoperative pain and functional recovery after cesarean delivery [13].

The impact of various fascial closure techniques on postoperative outcomes has been particularly documented for general and emergency surgery. Several randomized controlled trials and meta-analyses show that the interrupted and modified Smead–Jones techniques, compared to the continuous closure technique, promote better load distribution across the fascial incision, reduce localized mechanical stress, and therefore, reduce the risk of at wound complication [4,5,6,7,8,9]. van ‘t Riet et al. concluded that while there may not be a significant difference in overall wound integrity for a variety of closures, small differences in closure techniques may affect postoperative pain and patient comfort [5]. Our studies apply these insights to obstetric surgery, suggesting that better fascial alignment, through the closure technique of guide-suture-assisted modified retention, may decrease early postoperative pain and not add to the complexity of the procedure.

The current research has shown that patients who underwent guide-suture-assisted modified fascial closure reported lower scores on pain measurement scales (VAS, NRS, and SF-MPQ) in the early postoperative period. The noted differences in the measured pain scores were most significant in the first 24 h after surgery. This time frame is critical; higher pain scores in this time frame have been shown to be correlated with delayed recovery and the development of chronic pain [3,14]. Borges et al. noted that early postoperative pain as a predictor of an extended recovery period and subsequent increase in pain relief medication is noteworthy [14]. Similar to this, in our study, patients in the classical fascia closure group reported higher pain scores and required stronger analgesics more frequently, while patients in the modified closure group needed less analgesics and had better functional recovery [15]. Inter-individual variability in metabolic response to spinal anesthesia and analgesic agents may have influenced pain perception and quantitative pain scores, particularly within the first 24 postoperative hours, and should be considered as a potential limitation.

Functional recovery, particularly the mobilization component, is a hallmark in the enhanced recovery after surgery (ERAS) frameworks following cesarean sections. Early ambulation is associated with a decrease in thromboembolic event risk, improved respiration, and overall recovery and rehabilitation [2,16]. Veef and Van de Velde noted that analgesia post-cesarean should be constructed in a way that allows for early mobilization and lessens the adverse effects of opioids [16]. In this regard, the modified fascia closure group having significantly less pain, and consequently, greater walking distances at 12, 24, and 48 h post-surgery, helps reinforce the relevance of clinically observed pain reduction, leading to greater functional outcomes. Although improved mobility is beneficial, increased early mobilization may theoretically raise concerns regarding wound integrity, including a potential risk of dehiscence; however, no such complications were observed in the present study.

In low and middle-income settings, clinically addressing pain post-cesarean section is especially problematic due to the limited availability of multimodal analgesia and other pain control regimens. Kintu et al. and Hussen et al. noted postoperative pain that was poorly controlled and associated with a lack of mobilization, contributing to negative outcomes for the mother [13,15]. Our results suggest, even in the absence of intricate, complex analgesia, that a low-cost, single modification to surgical technique can lead to improved postoperative pain and early mobilization, especially in cesarean sections, thus addressing, in a meaningful manner, a worldwide surgical problem.

The qualitative aspect in a mixed-method study helps to provide more insights into the findings in the quantitative section. Most patients in the classical fascia closure group described pain as sharp, burning, or pulling, as well as fear of movement and being dependent on strong painkillers. In comparison, patients who underwent modified fascial closure described their pain as moderate, more manageable, and less anxiety-inducing. They demonstrated more confidence in walking soon after surgery and needed less pain medication. These experiences from the patients correlated well with the quantitative pain and walking data and validated the modified fascial closure technique to be beneficial from a patients’ perspective.

Recent bibliometric analyses have also recognized the significance of these findings. Zhai et al. highlighted a surge in the number of publications focused on managing pain after cesarean sections, with a primary focus on the use of analgesics and anesthesia, while other surgical technique modifications still remain largely unexplored [17]. By using a mixed-methods approach to evaluate the guide-suture-assisted modified fascial closure technique systematically, we have addressed a critical literature void and provided new evidence to support the need for surgical modifications in managing pain after a cesarean section. From a clinical perspective, this technique may be particularly beneficial in patients undergoing repeat or high-risk cesarean deliveries, where rapid recovery and early mobilization are critical.

Future studies should aim to evaluate the effectiveness of this modified fascial closure technique in women with a history of recurrent cesarean deliveries. Such investigations may provide further insight into the clinical applicability and long-term benefits of this technique in more complex obstetric populations.

### Limitations

This study has several limitations. First, it was conducted at a single center with a relatively limited sample size, which may restrict the generalizability of the findings. Second, postoperative outcomes were evaluated within a short follow-up period, precluding assessment of long-term pain and chronic pain development. Additionally, inter-individual variability in analgesic response and the absence of long-term wound integrity assessment may limit the interpretation of early postoperative outcomes. Finally, although surgical and anesthetic protocols were standardized, unmeasured individual pain perception and psychosocial factors may have influenced postoperative pain reporting.

## 5. Conclusions

This mixed-methods study demonstrates that a guide-suture-assisted modified fascial closure technique is associated with reduced early postoperative pain and improved functional recovery following cesarean section. Compared with the conventional continuous closure method, the modified technique resulted in lower pain scores, decreased reliance on strong analgesics, and earlier mobilization during the critical postoperative period.

From a clinical perspective, optimizing fascial closure represents a simple, low-cost, and easily applicable surgical modification that can meaningfully enhance postoperative comfort and recovery without increasing operative complexity. Incorporating such techniques into routine cesarean section practice may contribute to improved maternal outcomes, support early ambulation, and potentially reduce the risk of prolonged or chronic postoperative pain.

## Figures and Tables

**Table 1:** Demographic and baseline clinical characteristics of patients according to surgical technique.

Variable	Classical Fascia Closure (n = 30)	Modified Fascia Closure (n = 30)	*p*
Age (years), Mean ± SD	25.33 ± 3.86	26.83 ± 4.12	0.152
BMI (kg/m^2^), Mean ± SD	27.51 ± 1.39	27.43 ± 1.50	0.825
First mobilization time (hours), Median (IQR)	6 (5–8)	6 (5–8)	0.256

**Table 2:** Comparison of postoperative pain scores and walking performance between the classical and modified fascia closure techniques.

Scores	Classical Fascia Closure (n = 30)	Modified Fascia Closure (n = 30)	*p* Value
VAS Scores			
Postoperative 6th hour	7 (4–7)	5 (4–5)	0.010 *
Postoperative 12th hour	5 (4–6)	4 (3–5)	0.027 *
Postoperative 24th hour	3 (2–5)	2 (2–4)	0.031 *
Postoperative 48th hour	1 (1–3)	1 (1–2)	0.198
NRS scores			
Postoperative 6th hour	7 (4–7)	5 (4–6)	0.009 *
Postoperative 12th hour	5 (3–5)	4 (2–5)	0.007 *
Postoperative 24th hour	4 (2–6)	3 (2–4)	0.008 *
Postoperative 48th hour	3 (0–4)	2 (1–4)	0.352
SF-MPQ scores			
Postoperative 6th hour	6 (4–6)	5 (4–5)	0.014 *
Postoperative 12th hour	5 (3–7)	4 (3–4)	0.010 *
Postoperative 24th hour	3 (2–7)	2 (2–3)	0.021 *
Postoperative 48th hour	1 (1–3)	1 (1–2)	0.323
Resting VAS			
6th hour	5 (4–6)	4 (2–5)	0.006 *
12th hour	4 (3–5)	3 (2–4)	0.023 *
24th hour	3 (2–4)	2 (2–3)	0.005 *
48th hour	2 (1–2)	1 (1–2)	0.414
Ambulation VAS			
6th hour	7 (4–9	5 (3–9)	0.023 *
12th hour	6 (5–6)	4 (4–5)	0.024 *
24th hour	6 (5–6)	3 (1–5	0.018 *
48th hour	4 (2–4)	1 (1–2)	<0.001 *
Walking distance			
6th hour	10 (5–10)	12.5 (3–20)	0.114
12th hour	20 (10–20)	25 (15–60)	0.007 *
24th hour	40 (25–40)	100 (50–150)	<0.001 *
48th hour	100 (100–100)	152.5 (100–300)	<0.001 *

The data are presented as median (25th–75th percentile). Comparisons between groups were performed using the Mann–Whitney U test, and a p value < 0.05 was considered statistically significant. An asterisk (*) indicates statistically significant results.

**Table 3:** Comparison of postoperative analgesic use between the classical and modified fascia closure techniques.

Analgesic	Classical Fascia Closure (n = 30)	Modified Fascia Closure (n = 30)	χ^2^	*p* Value
Dexketoprofen trometamol	18 (60.0%)	30 (100.0%)	15.00	<0.001 *
Diclofenac sodium	30 (100.0%)	26 (86.7%)	44.35	<0.001 *
Tenoxicam	30 (100.0%)	26 (86.7%)	11.03	0.012 *
Tramadol hydrochloride	16 (53.3%)	5 (16.7%)	10.35	0.006 *
Paracetamol	18 (60.0%)	19 (63.3%)	0.37	0.830

* The data are presented as number of cases (percentage). Differences in the frequency of analgesic use between groups were compared using the chi-square test, and a p value < 0.05 was considered statistically significant.

**Table 4:** Themes, subthemes, codes, and representative quotations derived from qualitative content analysis.

Theme	Subtheme	Codes	Representative Quotations
**Perception of Pain**	Pain intensity	Sharp pain, pulling sensation, localized tension	“I felt a sharp pulling pain at the incision site, especially when I tried to move.” (Classical closure)
		Mild discomfort, tolerable pain	“It was more like discomfort than pain; I could tolerate it without much difficulty.” (Modified closure)
	Pain quality	Burning, stabbing	“The pain was burning and stabbing, particularly in the first hours.” (Classical closure)
		Pressure, tightness	“It felt more like pressure rather than actual pain.” (Modified closure)
**Mobilization Experience**	Ease of movement	Difficulty standing, fear of movement	“I was afraid to stand up because it felt like the stitches would tear.” (Classical closure)
		Confidence in movement	“I felt confident when standing up; it didn’t pull much.” (Modified closure)
	Walking ability	Limited steps, slow walking	“I could only take a few steps at first.” (Classical closure)
		Longer walking distance	“I was able to walk longer than I expected.” (Modified closure)
**Recovery Perception**	Analgesic adequacy	Need for strong analgesics	“Painkillers were necessary; otherwise, I couldn’t manage.” (Classical closure)
		Reduced analgesic need	“I didn’t feel the need for strong painkillers.” (Modified closure)
	Overall comfort	Discomfort at incision site	“The incision area constantly bothered me.” (Classical closure)
		Feeling comfortable	“Overall, I felt comfortable and more relaxed.” (Modified closure)

## Data Availability

The data presented in this study are not publicly available due to patient confidentiality and ethical restrictions. The dataset was prospectively collected by the authors at our clinic and is stored in institutional records. Data may be made available from the corresponding author upon reasonable request, subject to appropriate ethical approval and institutional permissions. This study was registered at ClinicalTrials.gov (Registration Number: NCT07436520).
